# CONQUER: an interactive toolbox to understand functional consequences of GWAS hits

**DOI:** 10.1093/nargab/lqaa085

**Published:** 2020-10-27

**Authors:** Gerard A Bouland, Joline W J Beulens, Joey Nap, Arno R van der Slik, Arnaud Zaldumbide, Leen M ’t Hart, Roderick C Slieker

**Affiliations:** Department of Cell and Chemical Biology, Leiden University Medical Center, 2333 ZA, Leiden, The Netherlands; Department of Epidemiology and Biostatistics, Amsterdam Public Health Institute, Amsterdam UMC, location VUmc, 1081 HV, Amsterdam, The Netherlands; Julius Center for Health Sciences and Primary Care, University Medical Center Utrecht, 3584 CG, Utrecht, The Netherlands; Department of Cell and Chemical Biology, Leiden University Medical Center, 2333 ZA, Leiden, The Netherlands; Department of Immunohematology and Blood Transfusion, Leiden University Medical Center, 2333 ZA, Leiden, The Netherlands; Department of Cell and Chemical Biology, Leiden University Medical Center, 2333 ZA, Leiden, The Netherlands; Department of Cell and Chemical Biology, Leiden University Medical Center, 2333 ZA, Leiden, The Netherlands; Department of Epidemiology and Biostatistics, Amsterdam Public Health Institute, Amsterdam UMC, location VUmc, 1081 HV, Amsterdam, The Netherlands; Molecular Epidemiology section, Department of Biomedical Data Sciences, Leiden University Medical Center, 2333 ZA, Leiden, The Netherlands; Department of Cell and Chemical Biology, Leiden University Medical Center, 2333 ZA, Leiden, The Netherlands; Department of Epidemiology and Biostatistics, Amsterdam Public Health Institute, Amsterdam UMC, location VUmc, 1081 HV, Amsterdam, The Netherlands

## Abstract

Numerous large genome-wide association studies have been performed to understand the influence of genetics on traits. Many identified risk loci are in non-coding and intergenic regions, which complicates understanding how genes and their downstream pathways are influenced. An integrative data approach is required to understand the mechanism and consequences of identified risk loci. Here, we developed the R-package CONQUER. Data for SNPs of interest are acquired from static- and dynamic repositories (build GRCh38/hg38), including GTExPortal, Epigenomics Project, 4D genome database and genome browsers. All visualizations are fully interactive so that the user can immediately access the underlying data. CONQUER is a user-friendly tool to perform an integrative approach on multiple SNPs where risk loci are not seen as individual risk factors but rather as a network of risk factors.

## INTRODUCTION

In the past decades, numerous genome-wide association studies (GWASs) have been performed to understand the genetic contribution of traits. While GWASs have provided valuable insight into putative mechanistic pathways, the way the identified risk loci exert their effect on traits remain largely unclear. In general, GWAS-associated loci are not limited to coding regions but are frequently found in intergenic regions (1). As such, inferring how risk loci jointly influence genes and their downstream pathways remains often unclear. To increase the understanding of those variants, an integrative approach is required where the effects of variants are investigated at a multitude of molecular levels.

In recent years, the number of rich publicly available datasets and repositories has tremendously increased, which include histone modification data, multi-tissue gene expression data, chromosomal interactions driven by initiatives such as GTEx and Epigenomics Roadmap. In addition, an increasing number of studies have investigated the relation between genetic variation and molecular measures, for example gene expression (eQTLs), lipids (lQTLs), metabolites (mQTLs) and proteins (pQTLs). These datasets provide a valuable resource for understanding the possible functional consequences of GWAS risk loci. Extracting, combining and analyzing relevant biological information from public datasets can be complicated and time-consuming. There are several tools to perform gene set enrichment analysis (2,3), colocalization (3), or tools to investigate a single SNP in disease-specific context such as the different Knowledge Portals based on HuGeAMP that exist for multiple diseases (4). Existing tools are often online, provide static plots or one type of analysis, rely on proprietary software, require GWAS summary statistics, miss intuitive user experience or contain outdated data and genome builds. For example, MAGENTA, a commonly used tool, was last updated in 2011 and is based on MATLAB (5).

A flexible offline all-in-one tool, where one can do pathway enrichment, colocalization analyses, compare a single or set of SNPs against an up-to-date compendium of QTLs and genomic data is currently lacking. To provide researchers with an easy to use interface with the latest data to comprehend the effects of variants, we developed an R-package named CONQUER (‘COmprehend fuNctional conseQUencEs R’). Given a single SNP or multiple SNPs associated with a disease or trait, CONQUER allows the user to efficiently extract relevant biological information from various repositories/databases. On these data, pathway enrichment can be performed in up to 44 tissues. Moreover, one can investigate a single SNP in more detail by comparing it to chromatin state segmentations, chromosomal interactions, expression-, lipid-, protein-, metabolite-, DNA methylation QTLs and perform Bayes Factor colocalization analysis to identify the causal variant. All these data are accessible through interactive visualizations in the local web browser.

## MATERIALS AND METHODS

*CONQUER* was developed in R version 4.0.2 and JavaScript Data-Driven Documents (d3.js) version 4.13.0. The data acquired for *CONQUER* are based on the human genome reference build GRCh38/hg38. The data are both collected from static sources and Application Programming Interfaces (APIs). The static sources are available in a separate R data package called *conquer.db*, which is loaded when required. As *conquer.db* is a separate package it is easily updated with the latest datasets without altering the programming structure of CONQUER. Static data include chromatin interactions, chromatin state segmentations, expression data, transcription factor binding sites, protein QTLs (pQTLs), lipid QTLs (lQTLs), splicing QTLs (sQTLs), DNA methylation QTLs (meQTLs) microRNA QTLs (miQTLs) and metabolite QTLs (mQTLs). Data of pQTLs (6–9), meQTLs (10), lQTLs (11), mQTLs (12,13), miQTLs (14–17) were acquired from their corresponding references. sQTLs were obtained from GTEx v8 and included as a static resource in *conquer.db*. The chromatin interactions were obtained from the 4D genome database (18). To have data from multiple tissues (*N* = 31), only IM-PET data were included in CONQUER. Originally these data were based on the human genome reference build GRCh19/hg19. UCSC LiftOver tool (19) was used to lift over the data to GRCh38/hg38. Chromatin state segmentations were obtained from the Roadmap Epigenomics Project for all cell types available (*N* = 127, 15-state model) (20). Normalized (TPM, Transcript per Million) expression data of all available tissues (*N* = 54) was obtained from GTEx v8 (21). Missing expression values were imputed with *k*-nearest neighbor and default parameters of the *impute.knn* function from the R-package impute (22).

The remaining data (linkage disequilibrium, gene information, eQTLs) are obtained from APIs. Elementary information about the SNP of interest is acquired from the Ensembl API (23). The linkage disequilibrium (LD) structure originates from the LDlink API (24). For both the Ensembl API and LDlink API the population can be specified, by default the population is set on Utah Residents with Northern and Western European Ancestry (CEU) from the 1000 Genomes Project phase 3 (25). The eQTLs and eGenes corresponding to the SNP of interest are computed making use of GTEx API v8 (21). By default, GTEx has an eQTL mapping window of one Mb upstream and downstream of the transcription start site of a gene. In *CONQUER*, we expanded the search space by including genes that have chromosomal interaction on the same chromosome with the LD region (*R*^2^ ≥ 0.80) of the leading SNP. For every queried SNP, *CONQUER* generates an *RData* output object containing all previously described data and stores it in a directory the user has provided.

For colocalization, pre-calculated eQTLs are obtained from GTEx (v8). Bayes Factor colocalization analysis was performed using the R-package *coloc* based on the normalized effect size and variance (26). Given that *CONQUER* may also identify new eQTLs, outside the standard one Mb window from the transcription start site used by GTEx, individual colocalization analysis can be performed on a single gene and tissue.

Interactive figures were made using JavaScript data-driven documents (d3.js) version 4.13.0, based on existing and newly developed plots. D3.js code was integrated in R making use of the htmlwidgets R-package (27) and all tools were integrated into the R package *CONQUER.d3*. Interactive heatmaps were made using plotly (28). The interactive Circos plot was made with the R-package BioCircos (29). Interactive tables were generated with the DT package (30).

## RESULTS

**Figure 1.**
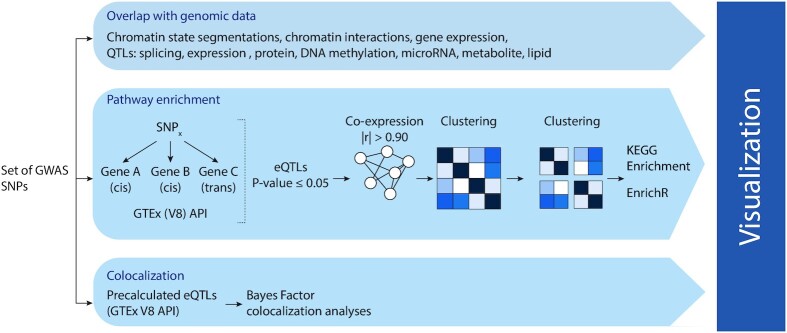
Overview of CONQUER. A set of SNPs is the input of CONQUER and is analyzed on three levels. First, SNPs are compared against a compendium of data, including multiple QTLs and other genomic data. Second, SNPs are tested for enrichment in KEGG pathways. In addition, the modules are tested for enrichment in number of databases using the R package enrichR, including Reactome, MSigDB. For this lead SNPs are tested against all genes in the proximity. The genes of significant eQTLs are then tested for co-expression. Co-expression matrices are clustered and cut in modules and on these modules, enrichment is performed. Only modules with one or more eQTLs are retained. Third, pre-calculated eQTLs are obtained and on these eQTLs Bayes Factor Colocalization analysis is performed to find the likely causal variant(s) for each gene in each tissue. Finally, the input of all three information sources is used as input for a shiny-based visualization dashboard.

CONQUER retrieves and interactively visualizes a multitude of public data associated with any single or set of independent SNPs of interest. The package can be used both for single and multiple SNPs. For the single SNP analysis there is no lower limit to the number of SNPs, but for the integrated analysis twenty SNPs and up is advisable to have enough eQTLs to perform the co-expression and enrichment on. There is no upper boundary, although >500 SNPs will take substantially longer to process. Of note, the pre-processing of the data can be performed in a cluster environment and the dashboard in a local environment. In case of single and multiple SNPs, CONQUER collects data about a SNP from multiple static and dynamic sources (see ‘Materials and Methods’ section, Figure 1). The user end of CONQUER consists of two intuitive function calls, *summarize*, and *visualize*. The summarize function minimally requires a list of SNPs (rs* IDs), a directory to store them in and an LDlink token to access the API and optionally a list of tissues. Finally, for each variant fine mapping is performed. For this pre-calculated SNPs are obtained from GTEx on which Bayes Factor colocalization analysis is performed. Summarize will collect the data and store data in small files per SNP and a separate file for the colocalization analysis.

To perform an integrated analysis of the SNPs, *multiAnalyze* should additionally be specified. CONQUER can modularize SNPs and associate them with biological pathways in tissues of interest (Figure 1). Based on the GTEx data, eQTLs and their associating eGenes are selected (*P*-value ≤ 0.05). Next, the eGenes and their co-expressed genes (Spearman's *ρ* ≥ |0.90|) are hierarchical clustered (31,32) based the distance between genes (*1 –* ρ). The number of modules within the clustered data is optimized by maximizing the globalSEmax of the gap statistic (33) using the *cluster* R package (34). Modules of co-expressed genes and eGenes are then tested for pathway enrichment based on KEGG pathways. For each pathway odds ratios and accompanying *P*-values are calculated with Fisher's exact test (35). If a module does not contain an eQTL or is not enriched for a pathway, it is omitted from the analysis. For the multi-tissue analysis, a separate file will be stored.

After summarization, the results can be visualized with the *visualize* function. It starts a Shiny-based dashboard, with interactive plots of the integrated analysis and a tab where individual SNPs can be investigated. Visualize requires the directory where the CONQUER files are stored and the SNP names to be included in the dashboard. CONQUER has two separate views (i) where in-depth analyses of single SNPs can be performed and (ii) where multiple SNPs associated with the trait of interest and their aggregated consequences can be investigated and linked to biological pathways. When multiple SNPs are analyzed, associated with a trait of interest, the first two tabs of CONQUER (*Modules* and *All SNPs)* give information about the relation between all investigated SNPs. The *Modules* tab shows on the tissue level the identified modules, the enriched pathways and the underlying SNPs and genes (Supplementary Figure S1), which can be further explored in more detail (Supplementary Figure S2). The *All SNPs* tab shows for each (non-) disease pathway in which tissue it was enriched. Moreover, it gives for all investigated SNPs including SNPs in LD, the known pQTLs, lQTLs, sQTLs, meQTLs, miQTLs and mQTLs.

**Figure 2.**
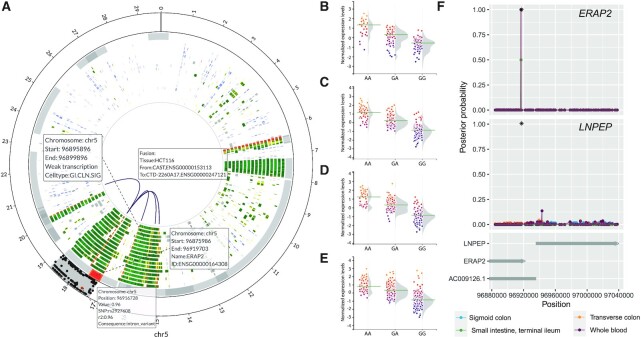
In depth analysis of the ERAP2 locus. (**A**) Circos plot of the *ERAP2* locus. From outer track to inner track: linkage disequilibrium, genes (gray), chromatin state segmentations, chromosomal interactions. The LD track shows in red the lead SNP from the GWAS. The black dots represent the SNPs in LD, with the *r*^2^ on the *y*-axis of the track. For the gene track, all known genes in the region are shown and hovering over genes gives information on the gene symbol, Ensembl ID, start and end of the gene. The chromatin state segmentations show information about the function of that region based on histone modifications. In the middle, the chromosomal interactions are shown. (**B**–**E**) QTL effect of rs1363907 on *ERAP2* expression in small intestine (B), transverse colon (C), sigmoid colon (D) and whole blood (E). (**F**) Bayes Factor colocalization analysis on *ERAP2*, *LNPEP* for the LD region of lead SNP for Crohn's disease, rs1363907. The black star indicates the location of rs1363907. *x*-axis, genomic location; *y*-axis, posterior probability of a SNP being the causal variant.

**Figure 3.**
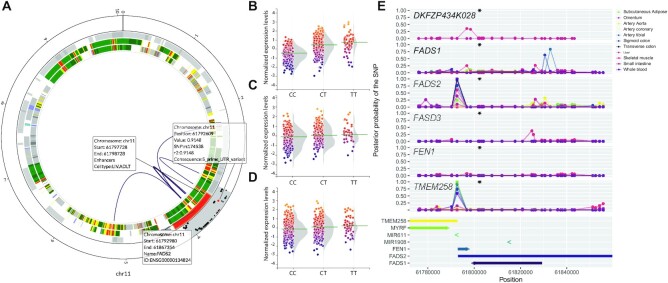
In depth analysis of the FADS2 locus. (**A**) Circos plot of the *FADS2* locus. (**B**–**D**) From outer track to inner track: linkage disequilibrium, genes (gray), chromatin state segmentations, chromosomal interactions. The LD track shows in red the lead SNP from the GWAS. The black dots represent the SNPs in LD, with the *r*^2^ on the *y*-axis of the track. For the gene track, all known genes in the region are shown and hovering over genes gives information on the gene symbol, Ensembl ID, start and end of the gene. The chromatin state segmentations show information about the function of that region based on histone modifications. In the middle, the chromosomal interactions are shown. (B–D) QTL effect of rs174546 on *FADS2* expression in whole blood (B), colon transverse (C) and tibial artery (D). (**E**) Bayes Factor colocalization analysis on *DKFZP434K028*, *FADS1, FADS2, FADS3, FEN1, TMEM258* for the LD region of rs174546. The black star indicates the location of HDL GWAS lead SNP rs174546. *x*-axis, genomic location; *y*-axis, posterior probability of a SNP being the causal variant.

The single SNP view (Supplementary Figure S3) is comprised of five tabs, that is *Linkage Disequilibrium*, *Chromosomal interactions*, *Chromatin States*, *QTLs* and *Gene expression*. The chromosomal interaction tab (Figures 2A and 3A) displays a circular view of the chromosomal region that contains genes, chromatin state segmentations and chromosomal interactions. The chromatin state segmentations of all tissues are displayed on a separate tab (Supplementary Figure S4). The QTL tab gives all afore mentioned QTLs for the selected SNP. In addition, the eQTL data are used to identify likely causal SNP(s) by using Bayes Factor Colocalization analysis. Finally, on the final tab the gene expression can be viewed of genes in the proximity of the LD region.

### Crohn's disease associated SNPs are enriched for natural killer cell mediated cytotoxicity

In order to demonstrate the utility of CONQUER, 165 SNPs associated with Crohn's disease (CD) were investigated in more detail in sigmoid and transverse colon, small intestine and in whole blood (36). As a benchmark, we ran the same set of SNPs in DEPICT (2). Between methods similar pathways were found (Supplementary Figure S5), including *Toll-like receptor signaling pathway* (CONQUER, Pc = 6.25·10^−9^; DEPICT, P_D_ = 1.59·10^−9^), *T-cell receptor signaling pathway* (Pc = 1.23·10^−8^; P_D_ = 2.93·10^−5^) in whole blood. Moreover all tissues, the pathway *Intestinal immune network for IgA production* was enriched in CONQUER (small intestine *P* = 3.35·10^−15^, sigmoid colon *P* = 5.00·10^−11^, transverse colon *P* = 1.77·10^−10^) and in DEPICT (P_D_ = 3.02·10^−6^). Toll-like receptors, T-cell and IgA signaling are known important processes in CD (37,38). In transverse colon (*P* = 9.89·10^−43^) and sigmoid colon (*P* = 2.45·10^−11^) the *Ribosome* pathway was strongly enriched (Supplementary Figure S5).

In addition, to expression QTLs and pathway enrichment CONQUER identifies QTLs on other levels. For the set of CD-associated SNPs, three SNPs were plasma pQTLs *in cis* for ERAP2 (rs1363907, *P* = 1.31·10^−6^), MST1 (rs3197999, *P* < 1·10^−16^) and IL18R1 (rs6708413, 9.00·10^−35^). Rs1363907 was also an eQTL for *ERAP2*(Figure 2A), i.e. terminal ileum of the small intestine (*P* = 2.1·10^−52^, Figure 2B), transverse colon (*P* = 1.40·10^−98^, Figure 2C) and sigmoid colon (*P* = 1.00·10^−81^, Figure 2D) and whole blood (*P* = 5.00·10^−177^, Figure 2E). ERAP2 is a protein known to be associated with immune-mediated diseases (39). Colocalization analysis revealed that not rs1363907, but rs2927608 was most likely the causal variant in both transverse colon and whole blood (posterior probability, PP = 1.00, Figure 2F). For the other two pQTLs, also eQTLs were identified. The variant near MST1 was an eQTL in sigmoid colon and transverse colon. The variant near IL18R1 was an eQTL in whole blood (*P* = 5.5·10^−7^), but not on the sigmoid colon and transverse colon. For both variants colocalization analysis did not point to a specific causal variant.

### SNPs associated with HDL cholesterol link to PPAR signaling and fatty acid-related pathways

As a second example, 71 SNPs associated with HDL (40) were investigated in adipose tissue, arteries, liver, colon, muscle and whole blood. Again, pathway enrichment was compared to DEPICT (Supplementary Figure S6). Among the enriched pathways, pathways relevant for HDL were identified in CONQUER that were also enriched in DEPICT. For example, in multiple tissues *PPAR signaling pathway* was enriched Supplementary Figure S6). CONQUER identified other relevant pathways including *Fatty acid degradation* in subcutaneous adipose fat (*P* = 3.63·10^−4^) and skeletal muscle (6.59·10^−3^) and *Ribosome* in subcutaneous- and visceral fat, skeletal muscle, small intestine and whole blood (*P* < 1.35·10^−11^).

Among the 71 SNPs, two were cis pQTLs and five lQTLs. For the latter rs174546 was associated with 31 different lipid species. The variant is located in the *FADS2* gene (Figure 3A), which is encodes fatty acid desaturase 2 confirming previous studies regarding this gene (41). In multiple tissues eQTLs were identified, including FADS2, including whole blood (*P* = 4.3·10^−54^, Figure 3B), colon transverse (*P* = 2.9·10^−16^, Figure 3C) and tibial artery (*P* = 4.8·10^−14^, Figure 3D). For *FADS2*, not rs174546 but rs174538 (r^2^ = 0.91 with rs74546) was the likely causal variant based on colocalization analysis in multiple tissues including whole blood (PP = 1.00), transverse colon (PP = 0.99) and tibial artery (PP = 0.96, Figure 3E). Rs174538 was also identified as the causal variant in the association with *TMEM258* in subcutaneous fat (PP = 1.00), tibial artery (PP = 0.92), sigmoid colon (PP = 0.74) and whole blood (0.59, Figure 3E). While rs174538 was not the lead SNP in the HDL GWAS used, it was the lead SNP in another study investigating the genetic influence on the omega-3 fatty acid eicosapentaenoic acid (41).

## DISCUSSION

In the current study, we have developed an R-package that aids researchers in understanding the functional consequences of SNPs. The R-package collects up-to-date data, directed by SNPs of interest from a multitude of databases and repositories, then, analyzes and visualizes the data. With the user-friendly and interactive dashboard, we were able to pinpoint SNPs and linked them to biological pathways in specific tissues. In contrast to previous studies that have had similar approaches (42,43), we have developed open-source software that is available as an R-package where only the SNPs and tissues of interest have to be specified.

Our package has several strengths. First, a large part of the package is based on APIs that automatically retrieve the latest data available, such as GTEx. Moreover, for GTEx CONQUER not only relies on the precalculated SNPs that are limited to one Mb around start sites of genes, but also calculates the relation between risk variants and more distant genes using GTEx's API. The other static resources that are included in CONQUER can be updated, given that they are stored in a separate package *conquer.db*. CONQUER will be maintained and updated at least twice a year to add new or updated data and make sure everything remains functional also when a new version of R is released. Second, CONQUER requires very basic programming experience and is implemented in the free open access software R. Third, CONQUER provides easy to understand and ready to publish visualizations that can be interactively explored in a web interface. Fourth, it provides not only information on the identified enrichments, but also allows researchers to investigate single variants in more detail, by looking at multiple types of associated QTLs, surrounding genomic regulation, genomic interactions and mRNA expression across tissues. Finally, CONQUER provides tissue-specific pathway enrichment. We benchmark CONQUER against DEPICT and we showed similar performance in both examples investigated, with the advantage of tissue-specific enrichment.

A limitation of the package is that the package is dependent on APIs which could be discontinued. However, it would require little adaptation to implement other APIs that provide the required information. This also applies to static sources CONQUER is built upon, that is when larger and better datasets become available these will be updated.

Together, our package is a user-friendly tool to perform an integrative approach on multiple SNPs where risk loci are not seen as individual risk factors but rather as a network. Moreover, one can in detail investigate single SNPs to find plausible mechanisms of action and fine map SNPs in LD to find the causal variant.

## DATA AVAILABILITY

CONQUER is available from Git (https://github.com/roderickslieker/CONQUER).

## Supplementary Material

lqaa085_Supplemental_Files
