# Supplementary material for: CONQUER: an interactive toolbox to understand functional consequences of GWAS hits
Source: NAR Genom Bioinform. 2020 Oct 27;2(4):lqaa085. doi: 10.1093/nargab/lqaa085 (PMC7671384; doi:10.1093/nargab/lqaa085)
Supplement: lqaa085_Supplemental_Files [file lqaa085_supplemental_files.zip › Figure_S5.pdf]

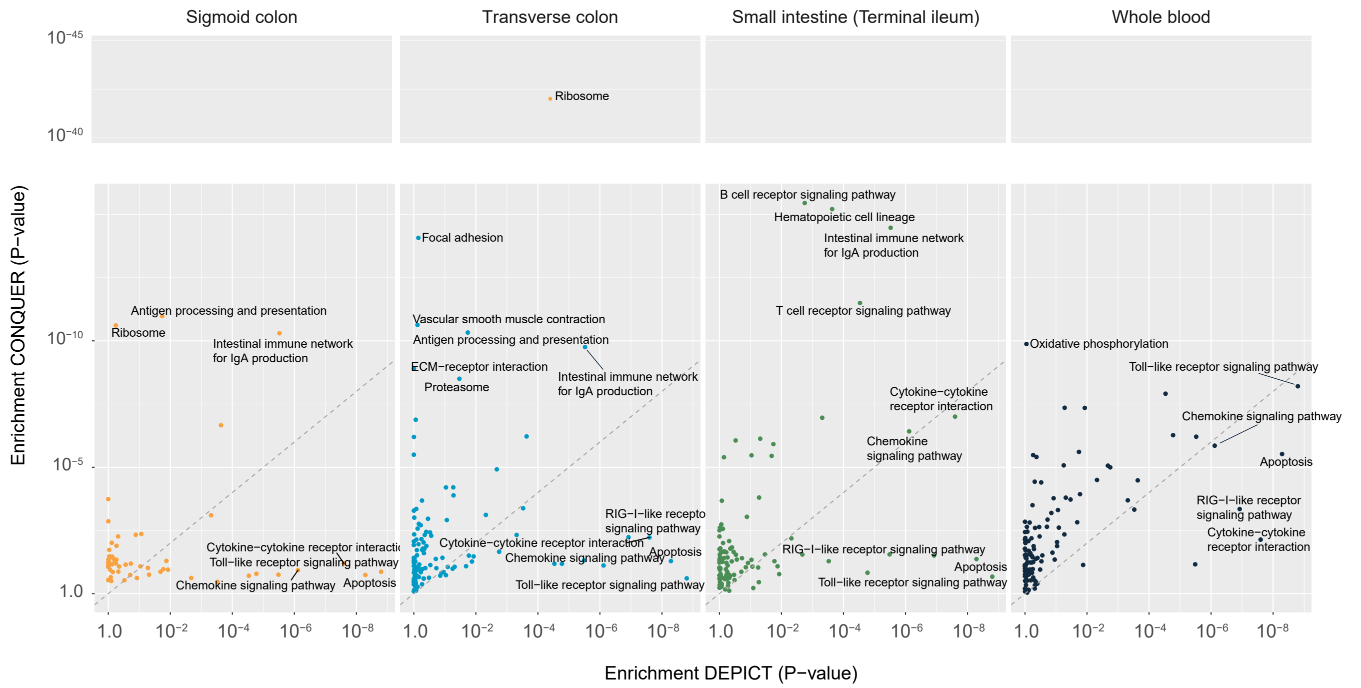
Sigmoid colon
Transverse colon
Small intestine (Terminal ileum)
Whole blood
Enrichment CONQUER (P−value)
Enrichment DEPICT (P−value)
Ribosome
Antigen processing and presentation
Intestinal immune network for IgA production
Cytokine−cytokine receptor interactio
Toll−like receptor signaling pathway
Chemokine signaling pathway
Apoptosis
Focal adhesion
Vascular smooth muscle contraction
ECM−receptor interaction
Proteasome
RIG−I−like recepto signaling pathway
Cytokine−cytokine receptor interaction
B cell receptor signaling pathway
Hematopoietic cell lineage
T cell receptor signaling pathway
Cytokine−cytokine receptor interaction
Chemokine signaling pathway
RIG−I−like receptor signaling pathway
Oxidative phosphorylation
RIG−I−like receptor signaling pathway
