# Supplementary material for: CONQUER: an interactive toolbox to understand functional consequences of GWAS hits
Source: NAR Genom Bioinform. 2020 Oct 27;2(4):lqaa085. doi: 10.1093/nargab/lqaa085 (PMC7671384; doi:10.1093/nargab/lqaa085)
Supplement: lqaa085_Supplemental_Files [file lqaa085_supplemental_files.zip › List of Supplementary Files.docx]

**Figure S1**. Screenshot of CONQUER dashboard. On top the different tabs. Figure represents the relation between tissues, identified modules, SNPs and genes. Pathways and genes can be clicked on which will show more details, for example the involved eQTLs in a pathway.

**Figure S2**. In depth information about the modules and enriched pathways based on HDL-associated loci. Heatmap shows the correlation between co-expressed genes. On the right the enriched pathways with the odds ratio for module 12 in whole blood.

**Figure S3**. Single SNP view for rs174546. Linkage disequilibrium plot is shown with the correlation between genes, the recombination rate and nearby genes. Bottom table shows provides more detail on the SNPs in LD.

**Figure S4**. Chromatin state segmentations for rs174546. On the rows the different cell types and on the x-axis the genomic location. Colors refer to different functions based on histone modifications. TSS, transcription start site; Enh, enhancer, ZNF, zinc finger.

**Figure S5**. Comparison of DEPICT and CONQUER for Crohn’s disease-associated SNPs. For the different tissues, the P-values of enriched pathways are compared. X-axis, DEPICT P-value and y-axis CONQUER P-value.

**Figure S6**. Comparison of DEPICT and CONQUER for HDL-associated SNPs. For the different tissues, the P-values of enriched pathways are compared. X-axis, DEPICT P-value and y-axis CONQUER P-value.
