# Supplementary figures and images for: CONQUER: an interactive toolbox to understand functional consequences of GWAS hits
Source: NAR Genom Bioinform. 2020 Oct 27;2(4):lqaa085. doi: 10.1093/nargab/lqaa085 (PMC7671384; doi:10.1093/nargab/lqaa085)

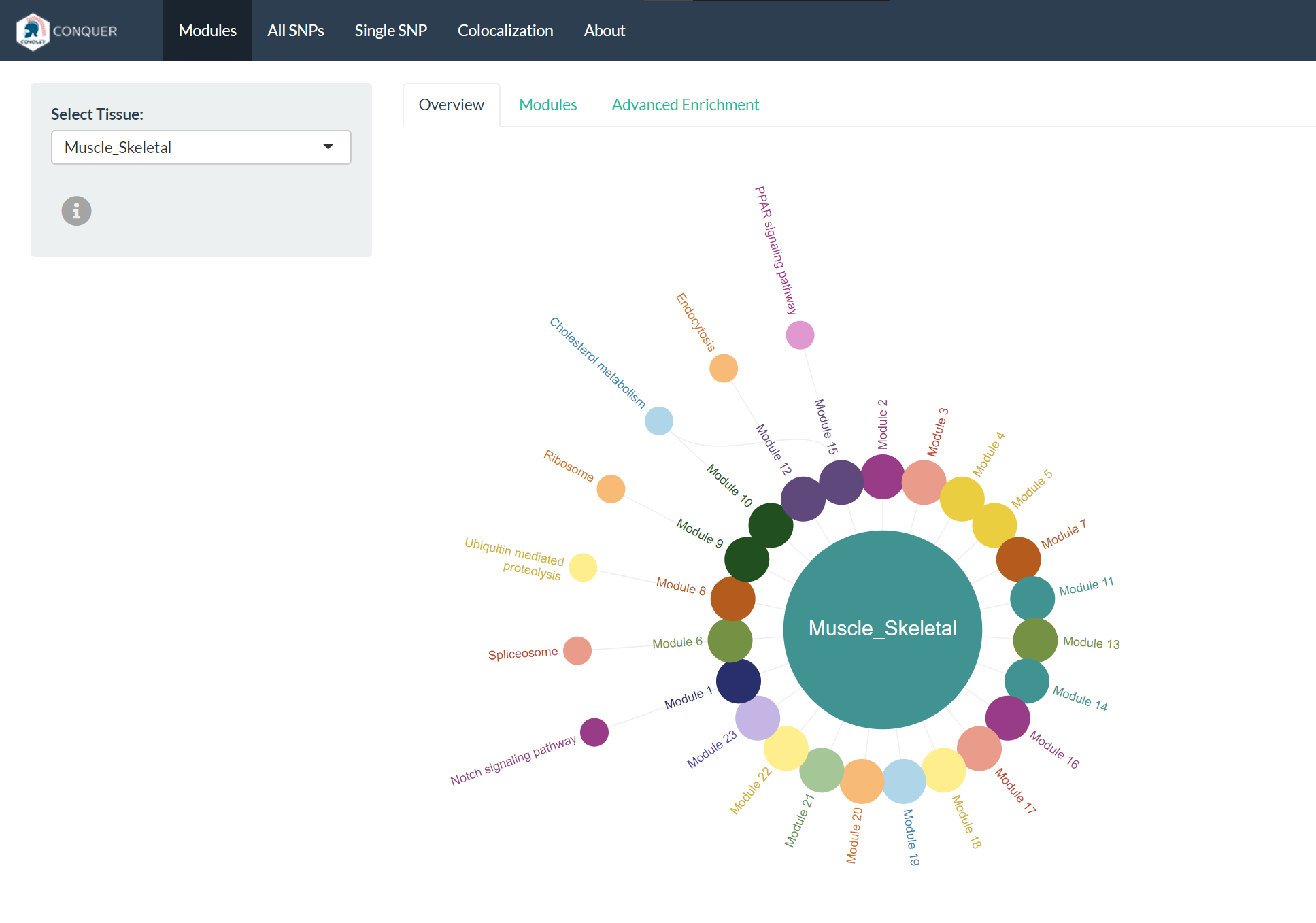

Supplement: lqaa085_Supplemental_Files [file lqaa085_supplemental_files.zip › Figure_S1.png]

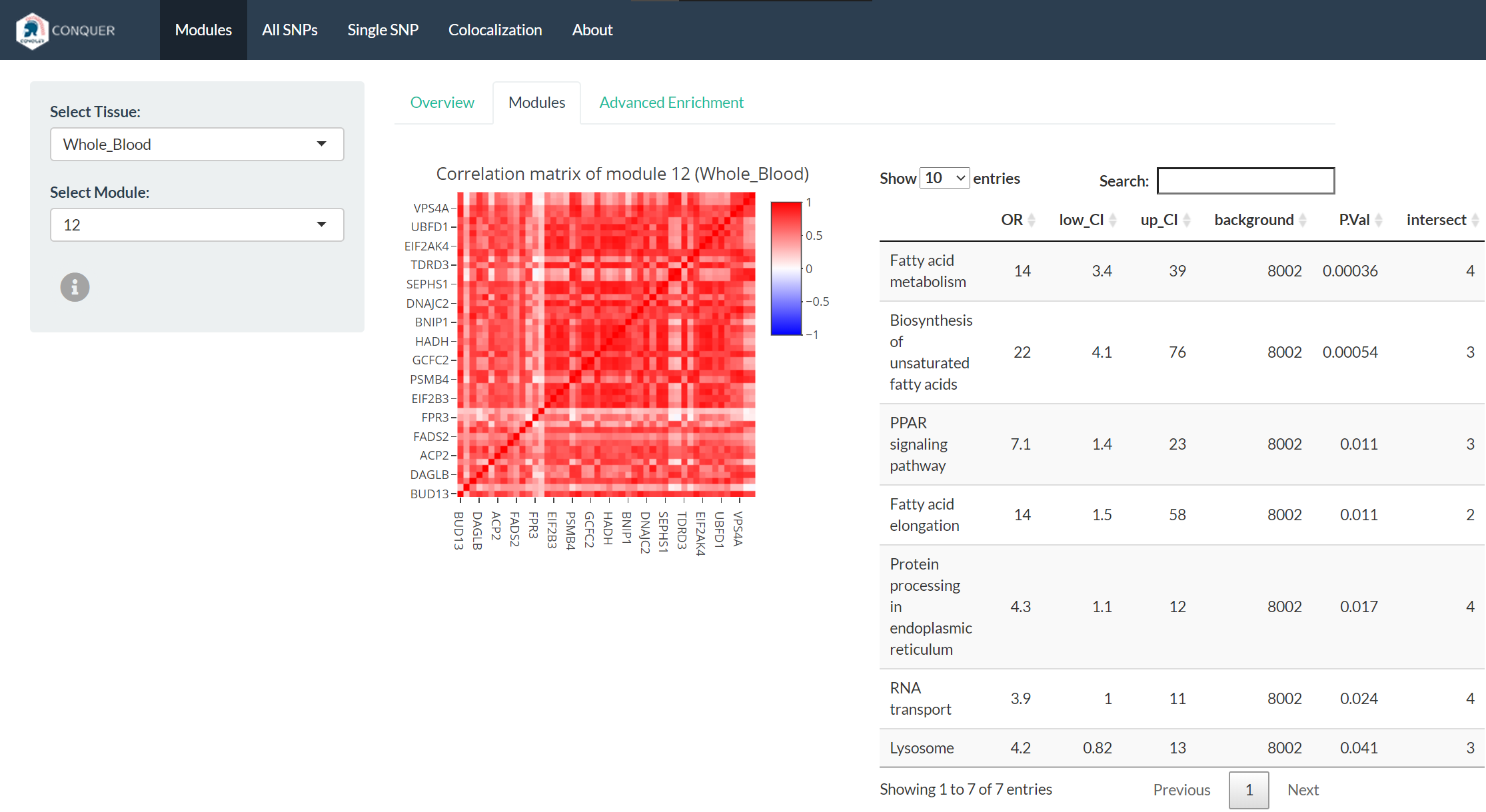

Supplement: lqaa085_Supplemental_Files [file lqaa085_supplemental_files.zip › Figure_S2.png]

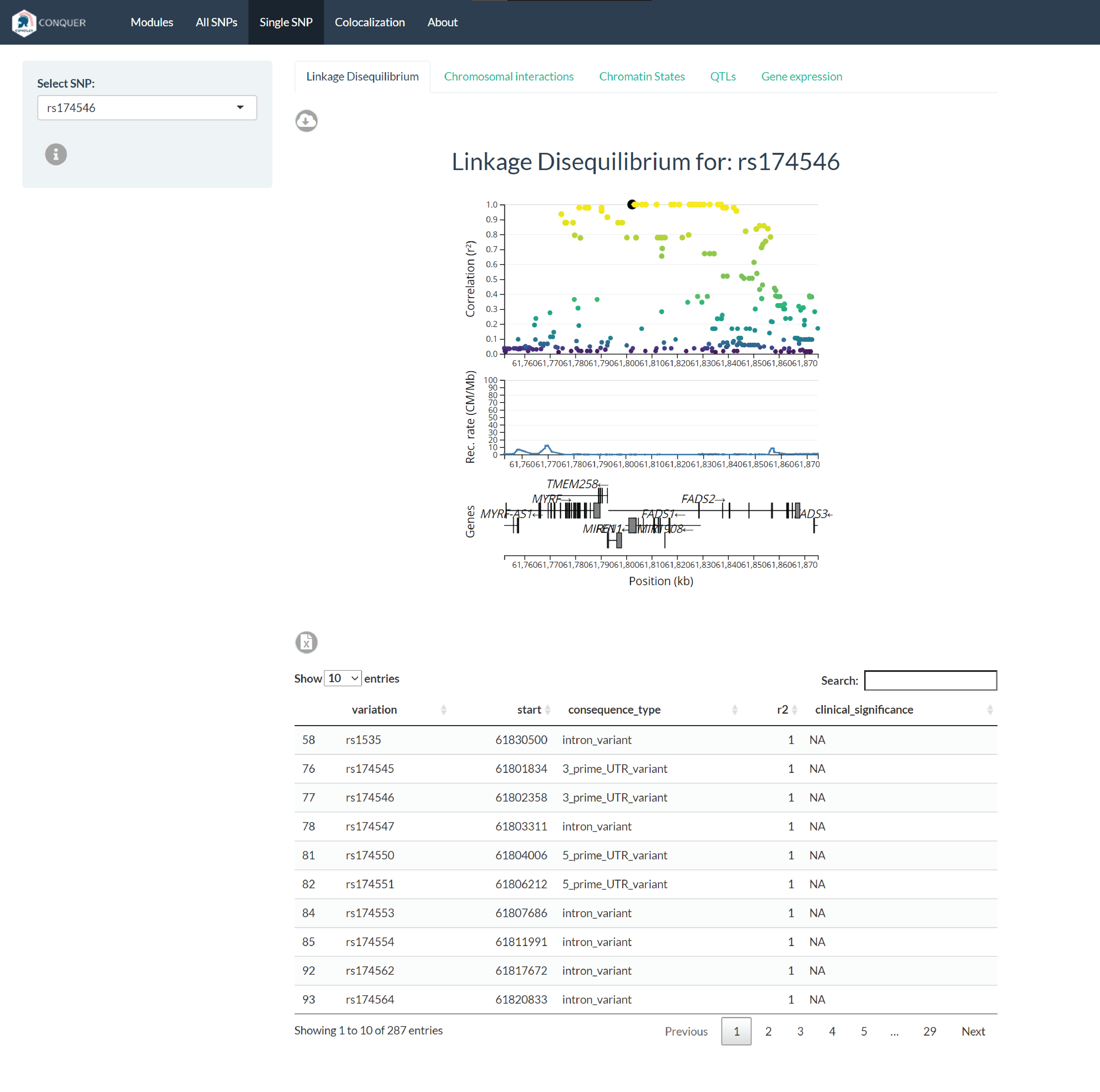

Supplement: lqaa085_Supplemental_Files [file lqaa085_supplemental_files.zip › Figure_S3.png]

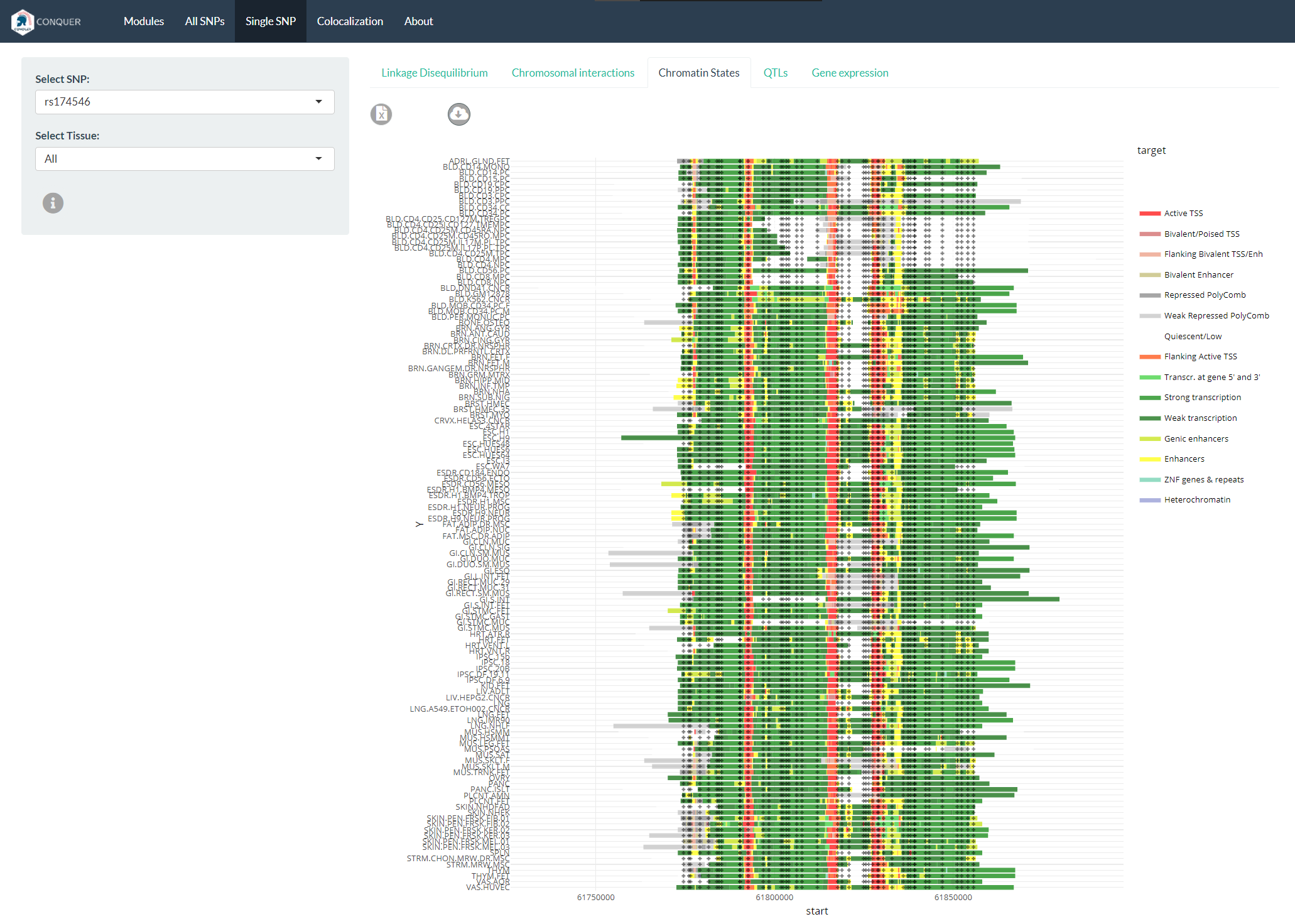

Supplement: lqaa085_Supplemental_Files [file lqaa085_supplemental_files.zip › Figure_S4.png]

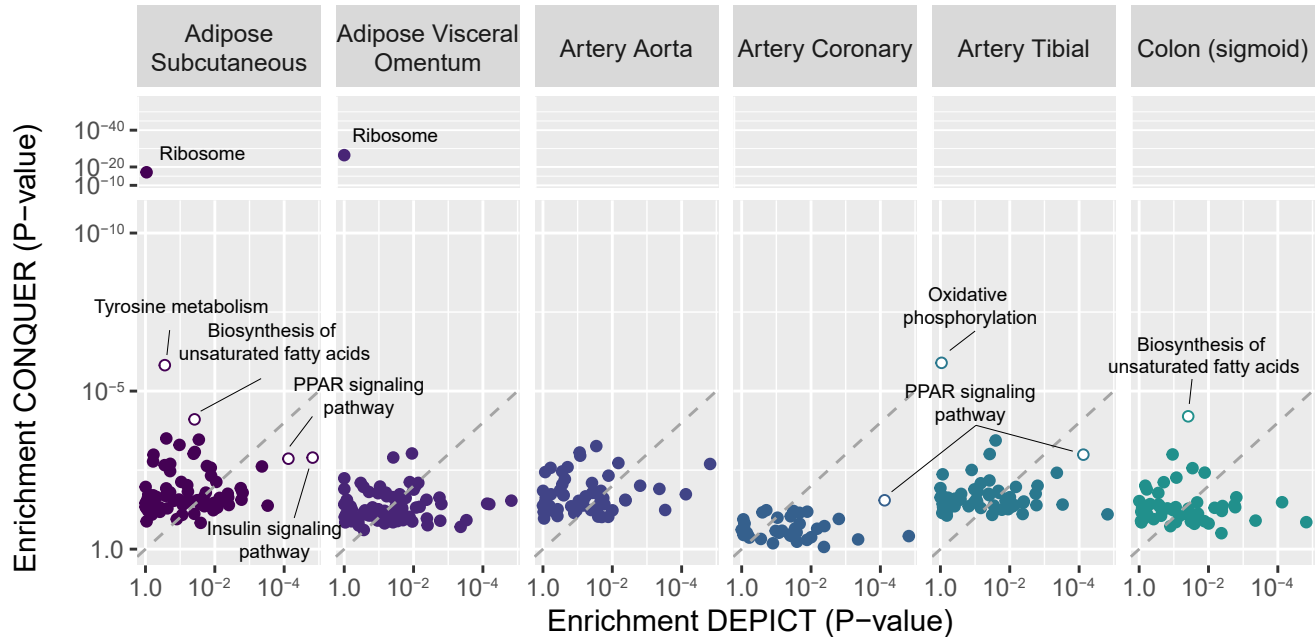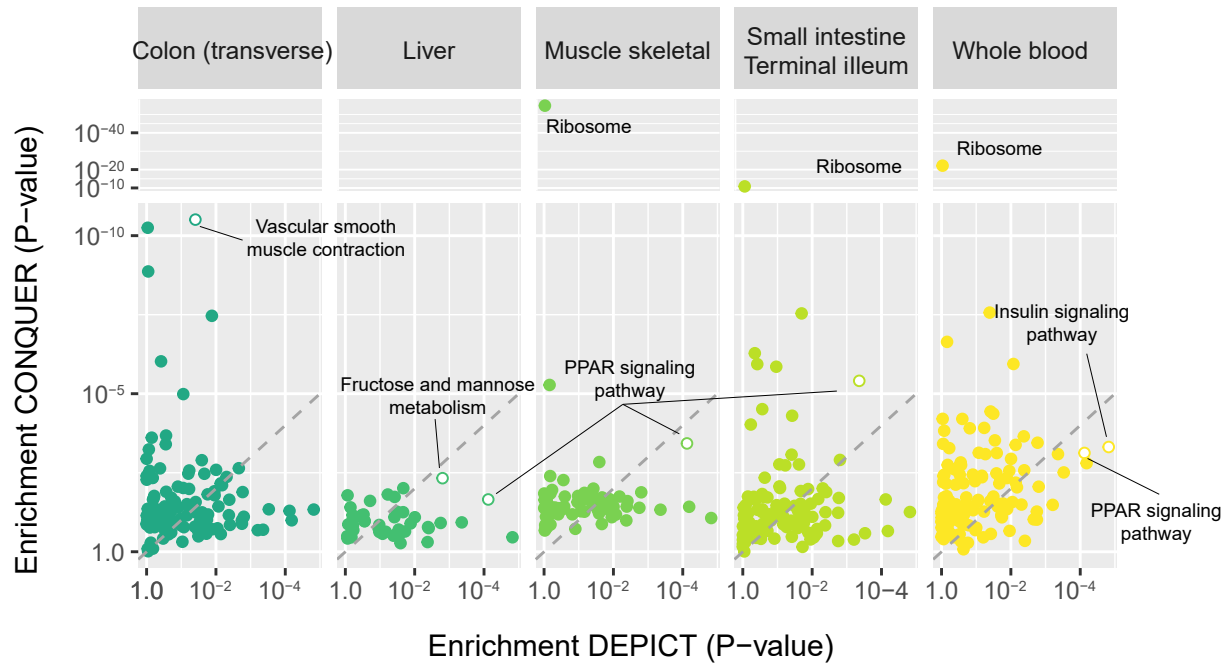

Supplement: lqaa085_Supplemental_Files [file lqaa085_supplemental_files.zip › Figure_S6.pdf]
